# Abnormal Cortical Thickness Is Associated With Deficits in Social Cognition in Patients With Myotonic Dystrophy Type 1

**DOI:** 10.3389/fneur.2020.00113

**Published:** 2020-02-28

**Authors:** Laura Serra, Guendalina Bianchi, Michela Bruschini, Giovanni Giulietti, Carlotta Di Domenico, Sabrina Bonarota, Antonio Petrucci, Gabriella Silvestri, Alessia Perna, Giovanni Meola, Carlo Caltagirone, Marco Bozzali

**Affiliations:** ^1^Neuroimaging Laboratory, IRCCS Fondazione Santa Lucia, Rome, Italy; ^2^UOC Neurologia e Neurofisiopatologia, AO San Camillo Forlanini, Rome, Italy; ^3^Department of Geriatrics, Orthopedic and Neuroscience, Institute of Neurology, Catholic University of Sacred Heart, Rome, Italy; ^4^Department of Neurorehabilitation Sciences, Casa Cura Policlinico, Milan, Italy; ^5^Department of Clinical and Behavioral Neurology, IRCCS Fondazione Santa Lucia, Rome, Italy; ^6^Brighton & Sussex Medical School, CISC, University of Sussex, Brighton, United Kingdom

**Keywords:** myotonic dystrophy type-1, social cognition, cortical thickness, MRI, emotions

## Abstract

**Aim:** To investigate the cortical thickness in myotonic dystrophy type 1 (DM1) and its potential association with patients' genetic triplet expansion and social cognition deficits.

**Methods:** Thirty patients with DM1 underwent the Social Cognition Battery Test and magnetic resonance imaging (MRI) scanning at 3 T. Twenty-five healthy subjects (HSs) were enrolled in the study to serve as a control group for structural MRI data. To assess changes in cortical thickness in DM1 patients, they were compared to HSs using a *t*-test model. Correlations were used to assess potential associations between genetic and clinical characteristics and social cognition performances in the patient group. Additionally, multiple regression models were used to explore associations between cortical thickness, CTG triplet expansion size, and scores obtained by DM1 patients on the Social Cognition Battery.

**Results:** DM1 patients showed low performances in several subtests of the Social Cognition Battery. Specifically, they obtained pathological scores at Emotion Attribution Test (i.e., Sadness, Embarrassment, Happiness, and Anger) and at the Social Situations Test (i.e., recognition of normal situation, recognition of aberrant behavior). Significant negative correlations were found between CTG triplet expansion size and Embarrassment, and Severity of Aberrant Behavior. Similarly, a negative correlation was found between patients' MIRS scores and Sadness. DM1 patients compared to HSs showed reduced thickness in the right premotor cortex, angular gyrus, precuneus, and inferior parietal lobule. Significant associations were found between patients' CTG triplet expansion size and thickness in left postcentral gyrus and in the left primary somatosensory cortex, in the posterior cingulate cortex bilaterally, and in the right lingual gyrus. Finally, significant associations were found between cortical thickness and sadness in the superior temporal gyrus, the right precentral gyrus, the right angular gyrus, and the left medial frontal gyrus bilaterally. DM1 patients showed a negative correlation between cortical thickness in the bilateral precuneus and in the left lateral occipital cortex and performance at the Social Situations Test. Finally, DM1 patients showed a negative correlation between cortical thickness in the left precuneus and in the superior frontal gyrus and scores at the Moral Distinction Test.

**Discussion:** The present study shows both cortical thickness changes in DM1 patients compared to controls and significant associations between cortical thickness and patients' social cognition performances. These data confirm the presence of widespread brain damages associated with cognitive impairment in DM1 patients.

## Introduction

Myotonic dystrophy type 1 (DM1) is an autosomal dominant neuromuscular disease (1). The genetic defect in DM1 results from the dynamic expansion of CTG repeats in the 3′ untranslated region of the dystrophia myotonica protein kinase (DMPK) region (1–3). Despite the prominent involvement of the muscular tissue remaining as the core feature of DM1, there is now substantial evidence that central nervous system abnormalities play a dominant role in accounting for highly impacting cognitive deficits in DM1 patients. Consistent evidence has been produced proving that structural and functional changes in DM1 brains account for some specific higher-level dysfunctions. So far, structural magnetic resonance imaging (MRI) studies have mainly focused on the assessment of regional gray matter volumetric and microstructural changes of the white matter tissue (4–12). Importantly, a strict relationship has been reported not only between structural brain abnormalities and DM1 patients' cognitive profile (e.g., executive functions, reasoning and visuospatial abilities) (7, 9) but also with patients' CTG triplet expansion (6, 8, 13). When looking at functional brain connectivity, abnormalities in specific networks have been identified to account for DM1 patients' personality traits (14), as well as for some complex cognitive impairments (8, 15) in the domain of social cognition (SC) (16). This is particularly relevant considering that higher-order neuropsychological dysfunctions, including SC, dominate the cognitive profile of DM1 patients who, conversely, perform relatively well on tests exploring single neuropsychological domains (e.g., memory, language, etc).

Social cognition involves different abilities such as Theory of Mind (ToM), emotion recognition and attribution, moral/non-moral judgments, decision making, and empathy. These abilities interact with each other to allow a socially appropriate behavior to be maintained in daily life (17). Deficits in any aspect of SC may result in an inappropriate behavior with different degrees of severity, depending on the extension of the underlying brain damage. Social cognition deficits have been consistently reported not only in patients with frontal brain lesions but also in patients with neurodevelopmental disorders such as autism or neurodegeneration such as the behavioral variant of the frontotemporal dementia (18). In DM1 patients, we recently identified functional brain abnormalities within networks implicated in ToM ability (16). Emotion recognition–wise, specific deficits have also been reported in patients with DM1 (19), although there are no available studies exploring their neurobiological substrate.

The aim of the present study was to investigate the neurobiological substrate of SC deficits in patients with DM1 by focusing on emotion attribution and moral/non-moral judgment abilities. Quantitative measures of cortical thickness were used here with the working hypothesis that focal brain changes might help understand these higher-level dysfunctions.

## Materials and Methods

### Participants

This study is part of broader research program that investigates the neural correlates of various aspects of SC in patients with DM1. We first investigated the association between ToM abilities and functional connectivity, as reported in Serra et al. (16). Then, we extended this investigation to emotion attribution, social situation, and moral–non-moral judgment abilities in a cohort of subjects that includes a large part of those already included in the former study (*n* = 26) (16), and we focused our investigation on the cortical thickness. In total, 30 patients with a molecular diagnosis of DM1 were recruited from the Neuromuscular and Neurological Rare Diseases Centre at San Camillo Forlanini Hospital (Rome, Italy) and from the Institute of Neurology at the Fondazione Policlinico Universitario A. Gemelli, IRCCS (Rome, Italy). In order to exclude the presence of a major cognitive impairment or mental retardation, patients had to be all suffering from a childhood, juvenile, or adulthood form of DM, as classified by the International Myotonic Dystrophy Consortium (IDMC) nomenclature (20), and they had to report a Mini Mental State Examination score (21) above 26. In addition, all patients had to show a normal level of intelligence quotient and no impairment in the comprehension ability, (16), anosognosia, or failures in school achievement. The CTG expansion size within the DMPK gene was assessed for all DM1 patients and used to classify them according to the IDMC nomenclature (2000). The Muscular Impairment Rating Scale (MIRS) (22) was used to clinically characterize the severity of muscle phenotype in DM1 patients. Twenty-five healthy subjects (HSs) were also recruited, at the Santa Lucia Foundation (Rome) and served as controls for MRI data analysis. They were recruited by public advertisement whose material was reviewed and approved by the local ethics committee. They were recruited from the same geographical areas of the patient group, and they had to be a native Italian speaker. As detailed below, all individuals showing an interest in taking part in the research underwent a medical screening to exclude the presence of any major neurological, psychiatric, or systemic disorder.

Principal demographic and clinical characteristics of the participants are summarized in Tables 1, 2. All participants were right handed as assessed by the Edinburgh Handedness Inventory (23). All subjects (patients and controls) underwent clinical assessment to exclude the presence of major systemic and neurological, psychiatric illnesses in controls, and pathologies different from known comorbidities in DM1 patients.

**Table 1:** Demographic and clinical characteristics of participants.

	**DM1 patients** ***n* = 30**	**HS** ***n* = 25**	***p*-value**
Mean (SD) age, y	41.9 (12.7)	39.0 (11.8)	0.385^a^
Gender (F/M)	15/15	13/12	0.882^b^
Mean (SD) years of formal education	12.2 (2.4)	16.3 (1.9)	0.0001^a^

*This table reports mean standard deviation (SD) and statistics (one-way analysis of variance or χ^2^) for demographic data (age, years of formal education, and gender distribution) in patients with DM1 and healthy subjects. DM1, myotonic dystrophy type 1; F, female; HS, healthy subjects; M, male*.

a *One-way analysis of variance*.

b *χ^2^*.

**Table 2:** Principal genetic and clinical characteristics of patients with DM1.

	**DM1 patients** ***n* = 30**
**Age at onset according with IDMC nomenclature:**	
Childhood or juvenile onset (age range, 6–19 y)	10 (33.3%)
Adulthood onset (age range, 20–60 y)	20 (66.6%)
**CTG triplet expansion (mean** **±** **SD) [range]**	527.6 ± 308.5 [73–1200]
**IDMC nomenclature**	
E1 (CTG range: 50–150), *n* (%)	2 (6.7%)
E2 (CTG range: 151–500), *n* (%)	15 (50.0%)
E3 (CTG range: 501–1,000), *n* (%)	11 (36.6%)
E4 (CTG range >1,000), *n* (%)	2 (6.7%)
**MIRS stage**	
Stage 2, *n* (%)	11 (36.6%)
Stage 3, *n* (%)	15 (50.0%)
Stage 4, *n* (%)	4 (13.3%)

*This table shows clinical onset, genetic features, and MIRS scores in DM1 patients. Frequencies and percentages are reported. DM1, myotonic dystrophy type 1; IDMC, International Myotonic Dystrophy Consortium; MIRS, Muscular Impairment Rating Scale*.

### Ethics Statement

This study was conducted according to the principles expressed in the Declaration of Helsinki. The ethics committee of the Santa Lucia Foundation approved the study. Written informed consent was obtained from all recruited subjects before study initiation.

### Social Cognition Assessment

All DM1 patients underwent the Social Cognition Battery developed by Prior et al. (24), which includes tests for the ToM, Emotion Attribution, Social Abilities, and Moral Judgments. For the purposes of the current study, we have reported here only data of Emotion Attribution, Social Abilities, and Moral Judgments. Performance scores obtained by DM1 patients at each test from the Social Cognition Battery were evaluated using the Italian normative cutoff reported by Prior et al. (24).

The associations between scores obtained at Social Cognition Battery and CTG triple's expansion, MIRS scores, and disease duration were assessed by using Pearson correlation coefficients.

### Image Acquisition and Preprocessing of Volumetric Images to Assess Cortical Thickness

All participants underwent MRI 3 T brain scanning (Siemens Medical Solutions, Erlangen, Germany) including the following acquisitions: (a) dual-echo spin echo (DE-SE) [repetition time (TR) = 5,000 ms, echo time (TE) = 20/100 ms]; (b) fast fluid-attenuated inversion recovery (FLAIR) [TR = 8,170 ms, TE = 96 ms, inversion time (TI) = 2,100 ms]; (c) 3D T1-weighted (TR = 7.92 ms, TE = 2.4 ms, TI = 210 ms, flip angle = 15°). For the dual-echo and FLAIR scans, 52 contiguous interleaved axial slices were acquired with a 2-mm slice thickness, with a 192 × 256 matrix over a 256 × 256-mm field of view, covering the whole brain. The T1-weighted volumes were acquired in a single slab, with a sagittal orientation and 224 × 256 matrix size over a 256 × 256-mm^2^ field of view, with an effective slice thickness of a 1 mm.

The T1-weighted images were automatically preprocessed for surface-based morphometry by using the pipeline included in CAT-12 (Computational Anatomy Toolbox 12), a toolbox of SPM 12 (https://www.fil.ion.ucl.ac.uk/spm/software/spm12/). Briefly, a projection-based thickness estimation was used to compute the cortical thickness and the central surface (25), including the partial volume correction, the sulcal blurring, and asymmetries corrections. CAT-12 permits to repair topological defects, using a method based on spherical harmonics (26) and to reparameterize the surface mesh into a common coordinate system using a specific algorithm to reduce the area distortion (27). Then, an adapted two-dimensional diffeomorphic DARTEL algorithm was used for the spherical registration of the brain surface. Finally, a smoothing with a Gaussian kernel of 15 mm (FWHM) was applied to each data set.

### Statistical Analyses

#### Demographic and Clinical Data

One-way analyses of variance were used to assess between group differences (DM1 vs. HS) in age and years of formal education. A χ^2^ test was used to test for differences in gender distribution (Table 1).

#### Social Cognition Battery

A series of Pearson coefficients were used to assess correlations between scores obtained by DM1 patients on the Social Cognition Battery and their CTG triplet expansions, and MIRS scores.

SPSS 21.0 (https://www.ibm.com/products/spss-statistics) was used to compute statistical analyses on demographic, clinical, and cognitive data.

#### Cortical Thickness Analyses

The MRI data analyses were performed in the framework of General Linear Model by using CAT-12 (Computational Anatomy Toolbox in SPM12) (https://www.fil.ion.ucl.ac.uk/spm/software/spm12/). A two-sample *t*-test model was used for voxel-wise comparisons of cortical thickness between DM1 patients and HS. Age, years of formal education, and intracranial volumes (obtained as sum of gray matter, white matter, and cerebrospinal fluid) were always used as covariates of no interest.

Moreover, only in patients with DM1 a series of multiple regression models were used to assess the potential association between cortical thickness and performance obtained at SC battery. Intracranial volumes were again entered as covariate of no interest together with CTG triplet expansions.

All results were accepted if survived at correction for multiple comparisons (*p* < 0.05 Family Wise Error (FWE) at cluster level).

## Results

As shown in Table 1, patients and controls did not differ significantly in age and gender distribution (*F* = 0.77, *p* = 0.385; χ^2^ = 0.02, *p* = 0.882) As expected, DM1 patients were significantly less educated than HSs (*F* = 42.6, *p* = 0.0001).

In DM1 group, 20 of 30 (66.6%) had an adulthood onset of disease, and 10 of 30 patients (33.3%) had childhood onset. Mean n(CTG) expansion size was 527.6 ± 308.5 (range, 73–1,200). According to the classification from of the Myotonic Dystrophy Consortium (20), 2 of 30 patients (6.7%) from our cohort of study belonged to E1 class, 15 of 30 (50.0%) to E2 class, 11 of 30 (36.6%) to E3 class, and finally 2 of 30 (6.7%) to E4 class. Based on MIRS rating, 11 of 30 (36.6%) had an MIRS score = 2, 15 of 30 (50.0%) had an MIRS score = 3, and 4 of 30 (13.3 %) had an MIRS score = 4.

### Social Cognition Assessment

All DM1 patients reported pathological scores in all items of the Social Situations Test and performed poorly on the Emotion Attribution Tests (Table 3). Remarkable deficits were observed for Sadness, Embarrassment, Happiness, and Anger.

**Table 3:** Social cognition battery performances obtained by patients with DM1.

**Social cognition battery**	DM1 patients^*^
**Emotion attribution test**	
Sadness mean (SD) cutoff ≥6	**5.63 (2.25)**
Fear mean (SD) cutoff ≥8	8.43 (1.04)
Embarrassment mean (SD) cutoff ≥8	**4.10 (3.59)**
Disgust mean (SD) cutoff ≥2	2.36 (0.99)
Happiness mean (SD) cutoff ≥10	**7.76 (1.81)**
Anger mean (SD) cutoff ≥6	**3.76 (2.34)**
Envy mean (SD) cutoff ≥1	2.16 (1.05)
**Social situations test**	
Recognition of normal situations mean (SD) cutoff ≥13	**12.68 (1.83)**
Recognition of aberrant behavior mean (SD) cutoff ≥22	**21.34 (2.24)**
Severity of Aberrant Behavior mean (SD) cutoff ≥45	**44.34 (8.23)**
**Moral/conventional distinction test**	
Moral behavior	
Behavior not permitted mean (SD) cutoff ≥6	6.0 (0.0)
Severity of aberrant behavior mean (SD) cutoff ≥39	53.52 (6.29)
Behavior not permitted with no rules mean (SD) cutoff ≥11	11.32 (1.65)
Conventional behavior	
Behavior not permitted mean (SD) cutoff ≥5	8.89 (2.43)
Severity of aberrant behavior mean (SD) cutoff ≥20	39.46 (10.6)
Behavior not permitted with no rules mean (SD) cutoff ≥6	5.39 (0.62)

*This table shows performance's scores (mean and standard deviation) obtained by DM1 patients in each test of the Social Cognition Battery (24). Italian normative cutoffs are reported. The patients' pathological performances are shown in bold font. DM1, myotonic dystrophy type 1*.

* *Performances scores in DM1 patients were evaluated using the Italian normative cutoff (24). Pathological performances are highlighted in bold characters*.

#### Relationship Between Performances at Social Cognition Battery and Genetics and Clinical Characteristics in DM1 Patients

Significant negative correlations were found between n(CTG) size in leukocytes and scores obtained at the Emotion Attribution Test (Embarrassment: *r* = −0.41, *p* = 0.03) and at the moral/non-moral Conventional Distinction Test (Severity of Aberrant Behavior: *r* = −0.37, *p* = 0.05). Negative correlation was also found between patients' MIRS scores and performances obtained at the Emotion Attribution Test (Sadness: *r* = −0.62, *p* = 0.002). Finally, no significant correlations were found between disease duration and SC scores.

### Cortical Thickness

#### Between-Group Comparison

**Figure 1:**
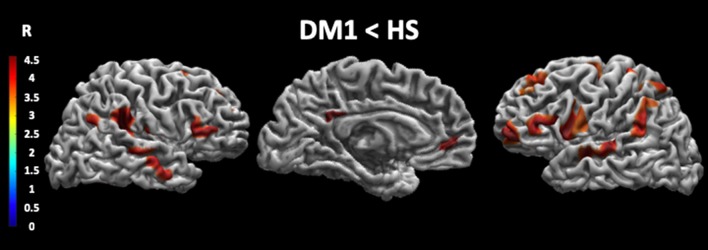
Cortical thickness in DM1 patients compared with HS. This figure reveals that patients with DM1 compared to healthy subjects showed a significant decrease in cortical thickness in several brain areas. In particular, the cortical thickness is significantly decreased in the precuneus, the angular gyrus, the superior temporal gyrus and the medial frontal gyrus bilaterally, the right precentral gyrus, the right posterior and anterior cingulate cortex, and the left superior parietal lobule. The statistical comparisons were overlapped on MRIcron ch2bet template (https://www.nitrc.org/projects/mricron). DM1, myotonic dystrophy type 1; HS, healthy subjects; R, right.

As shown in Figure 1 and Table 4, patients with DM1 compared to HSs showed a significant decrease in cortical thickness in several brain areas, including the precuneus, the angular gyrus, the superior temporal gyrus, and the medial frontal gyrus bilaterally; the right precentral gyrus and the right posterior and anterior cingulate cortex; and the left superior parietal lobule.

**Table 4:** Comparison between patients with DM1 and HSs in cortical thickness.

**Brain areas**	**Side**	**Size**	***Z***	**MNI coordinates**		
				***x***	***y***	***z***
Precuneus	R	350	3.99	8	−59	46
Angular gyrus	R	809	4.74	45	−50	35
Sup. temporal gyrus	R	179	4.16	55	−32	12
Precentral gyrus	R	155	4.09	23	−10	62
Fusiform gyrus	R	174	4.08	38	−50	−11
Inf. parietal lobule	R	120	3.88	47	−68	31
Med. frontal gyrus	R	143	3.83	49	16	32
Sup. frontal gyrus	R	160	3.80	21	17	53
Sup. temporal gyrus	L	502	4.89	−49	−41	19
Precuneus	L	324	4.69	−35	34	24
Med. frontal gyrus	L	681	4.51	−33	5	34
Paracingulate gyrus	L	234	3.97	−11	8	53
Angular gyrus	L	340	3.91	−51	−55	33
Precentral gyrus	L	214	3.76	−19	−19	67
Sup. parietal lobule	L	315	3.75	−27	−49	65
Sup. frontal gyrus	L	244	3.54	−14	17	64

*This table illustrates the brain areas in which DM1 patients show decreased cortical thickness compared to HS. Side, cluster size, statistic, and MNI coordinates are reported. DM1, Myotonic dystrophy type 1; HS, healthy subjects; Inf., inferior; L, left; Med., medial; MNI, Montreal Neurological Institute; R, right; Sup., superior*.

#### Relationship Between Cortical Thickness and CTG Triplet Expansion in Leukocytes

**Figure 2:**
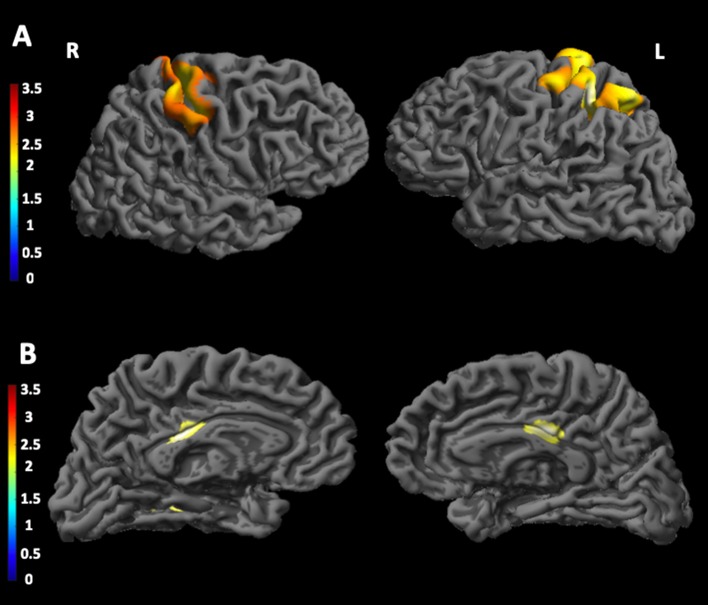
Correlations between CTG triplet expansion and cortical thickness in DM1 patients. The figure shows the correlations between CTG triplet expansion and the cortical thickness in different brain areas. In particular, **(A)** shows a significant positive correlation between CTG triplet expansion and cortical thickness in the left postcentral gyrus and in the left primary somatosensory cortex; **(B)** shows a significant negative correlation between CTG triplet expansion and cortical thickness in the posterior cingulate cortex bilaterally and in the right lingual gyrus. The statistical comparisons were overlapped on MRIcron ch2bet template (https://www.nitrc.org/projects/mricron). L, left; R, right.

As shown in Figure 2, we found significant positive correlation (A) between n(CTG) size in leukocytes and cortical thickness in the left postcentral gyrus and in the left primary somatosensory cortex. Conversely, a significant negative (B) correlation was found between patients' n(CTG) size in leukocytes and cortical thickness in the posterior cingulate cortex bilaterally and in the right lingual gyrus.

#### Relationship Between Cortical Thickness and Performances at the Social Cognition Battery

**Figure 3:**
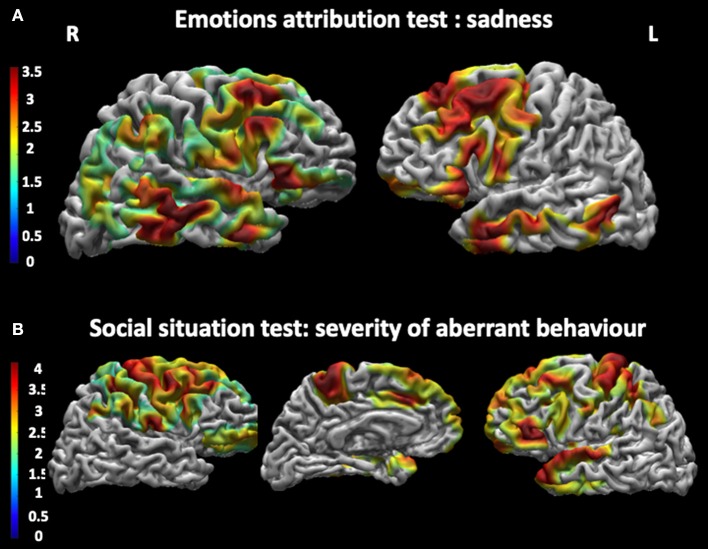
Correlations between cortical thickness and performances at the Social Cognition Battery. **(A)** shows a significant positive correlation between cortical thickness and correct attribution of Sadness in the Emotions attribution test in the superior temporal gyrus, the right precentral gyrus, the right angular gyrus and the left medial frontal gyrus bilaterally. **(B)** shows a negative correlation between cortical thickness in the bilateral precuneus and in the left lateral occipital cortex and performance at the Social Situations Test, especially in Severity of Aberrant Behavior. The statistical comparisons were overlapped on MRIcron ch2bet template (https://www.nitrc.org/projects/mricron). L, left; R, right.

As shown in Figure 3 and Table 5, patients with DM1 showed a significant positive correlation between correct attribution of sadness and cortical thickness in the left superior temporal gyrus, in the right inferior frontal gyrus, in the right precentral gyrus, in the right angular gyrus, and in the medial frontal gyrus bilaterally. They also showed negative correlations between performances at the Social Situations Test and cortical thickness in the bilateral precuneus, in the right superior parietal cortex, and in the left lateral temporal and occipital cortex.

**Table 5:** Relationship between cortical thickness and performances at the Social Cognition Battery in patients with DM1.

**Social Cognition Battery**	**Brain areas**	**Side**	**Size**	***Z***	**MNI coordinates**		
					***x***	***y***	***z***
Emotions attribution test: sadness	Sup. temporal gyrus	L	1577	3.73	−51	−4	−6
	Precentral gyrus/med. frontal gyrus	L	3264	2.97	−42	3	38
	Sup. temporal gyrus	R	2678	3.82	53	−10	−7
	Inf. frontal gyrus/precentral gyrus	R	5915	3.71	50	11	23
	Angular gyrus	R	966	2.95	43	−56	61
Social situations test: severity of aberrant behavior	Precuneus	L	5647	3.56	−18	−49	56
	Lat. Temporal cortex/ lat. occipital cortex	L	2425	3.23	−43	−44	−7
	Sup. parietal cortex/precuneus	R	9899	4.00	26	−56	56

*This table shows the statistically significant relationships between cortical thickness and performance' scores obtained by DM1 patients at Social Cognition Battery. Side, cluster size, statistic, and MNI coordinates are reported. DM1, Myotonic dystrophy type 1; Lat., lateral; L, left; Med., medial; MNI, Montreal Neurological Institute; R, right; Sup., superior*.

## Discussion

The present work shows a widespread impairment of SC in patients with DM1, involving Emotion Attribution, Social Situation, and Moral Judgment. Theory of Mind deficits were previously documented in DM1 patients and associated with abnormal functional connectivity in specific brain networks (16). More recently, face emotion recognition was found impaired in a cohort of DM1 patients with prominent difficulties for faces showing negative emotions such as anger and disgust, and crucially deficits were more extensive than expected (19). In our current work, we confirmed the conclusions drawn by Labayru et al. (19), and we contributed to clarify the neurobiological substrates of SC deficits in DM1 patients, especially for their emotion processing and moral/non-moral judgments abilities. We have indeed confirmed the presence of difficulties in emotion processing (mainly for negative emotions) with our patients scoring below the normality cutoff when required to recognize not only sadness, embarrassment, and anger, but also happiness. Nevertheless, our findings are also partially different compared to those reported by Labayru et al. (19), who found difficulties mainly in the recognition of anger and disgust. In our opinion, such differences are likely due to the different material used to assess emotions; indeed, Labayru et al. (19) used a facial emotion recognition test, whereas in our patients, we evaluated the emotion attribution processing using brief verbal stories. This test, which is part of the Social Cognition Battery, includes 58 brief stories that are read by the clinician. In each story, the protagonist is implicated in a social situation that causes a well-defined emotion (e.g., “Simon's pictures were ranking in the last in an artistic contest. How does Simon feel?”). This type of material is more cognitively demanding than facial visual stimuli and possibly more sensitive to minimal deficits. There were indeed no significant clinical differences between our own and Labayru's cohort of patients to justify different levels of SC impairment.

In the current study, we explored if any associations would exist between patients' emotion recognition abilities, severity of motor disability, or their CTG triplet expansion size in leukocytes. Consistent with our previous report on ToM deficits in DM1 (16), the current study confirms a significant association between patients' deficits in emotion recognition, their MIRS scores, and n(CTG) size in leukocytes. Within the uncertainty given by individual somatic mosaics, these data open a discussion about the relationship existing between n(CGT) in leukocytes and those segregating in specific cortical brain areas. It is possible that abnormalities in different domains of SC might be due to patients' genetic characteristics in addition to their mentalizing ability. The presence of somatic mosaicism has been traditionally demonstrated in the skeletal muscle, leukocytes, oocytes, and sperm of DM1 patients. Moreover, the size of repeat expansions is known to increase over time and across generations, thus causing somatic mosaicism also in postmitotic tissues, such as the skeletal and cardiac muscle and the brain (28–31). A recent postmortem study in DM1 patients has demonstrated the presence of a more remarkable somatic mosaicism in the brain tissue than in white blood cells. Remarkable expansion and somatic instability were present in all brain regions with the exception of the cerebellum (32). There are quite a few postmortem studies that have investigated the presence of molecular hallmarks of DM within the brain tissue. For instance, RNA nuclear foci were found being widely distributed throughout the cortical layers of various brain regions within different cell types, including neurons, astrocytes, oligodendrocytes, and Purkinje cells (33, 34). Moreover, RNA foci were found colocalizing with MBNL1 and MBNL2 proteins. Animal models, such as MBNL knockout mice and transgenic mice expressing high CTG repeats, have also been developed to further investigate these pathophysiological aspects of DM1. Interestingly, a direct comparison between MBNL1 and MBNL2 knockout mice revealed a major pathophysiological role for MBNL2 in the brain tissue (35). Additionally, the MBNL2 knockout model revealed altered REM sleep regulation, learning and memory deficits in association with GRIN 1 activity, and impaired long-term potentiation (36). The model of transgenic mice expressing more than 1,000 CTG repeats revealed the presence of numerous RNA foci within various brain regions alongside with an increase in CELF proteins in the brainstem and in the frontal cortex. These abnormalities were associated to spatial and working memory impairments and anhedonia, which are similar to the symptoms observed in human DM1 patients (34). Against this background, we can speculate that the abnormalities we observed in our patients might at least partially reflect this sort of pathophysiological mechanisms.

However, for a greater completeness, we report here that no significant correlations were found between SC scores and disease duration. This is not surprising considering that disease duration is calculated on the bases of the clinical onset of motor symptoms that do not necessarily reflect what occurs to the brain tissue.

The major novelty of the present study is the association found between cortical thickness and performances at the SC battery in patients with DM1. When comparing patients and healthy controls, the former showed a decrease in cortical thickness in several brain areas, including the precuneus and angular gyrus, the superior temporal gyrus, and the medial frontal gyrus bilaterally; the right precentral gyrus and posterior and anterior cingulate cortex; and the left superior parietal lobule. With the only exception of the precentral gyrus, all remaining regions are part of the association cortex and are known to play a key role in several higher-level functions. Therefore, they may be implicated also in determining the SC deficits that we observed in our DM1 patients. Conversely, the precentral gyrus is more implicated in planning, control, and execution of motor gestures. It has been previously considered as part of the mirror neuron system (37), and it plays an important role in different aspects of SC, such as mentalizing ability, empathy, and so on.

In addition, we found that changes in the cortical thickness in some of these areas were significantly associated with the peripheral patients' CTG triplet expansion size, thus supporting the idea that such cortical abnormalities could play an important role for the pathophysiology of emotional defects in DM1 patients. In particular, we found both positive and negative correlations between CTG triplet expansion size and cortical thickness. In several genetic conditions, such as Down syndrome (38), Williams syndrome (39), and Prader-Willi syndrome (40), but also in neurodevelopmental disorders such as autism (41), both increased and decreased gray matter volumes were found. In these studies, compensation brain mechanisms were hypothesized. Although the underlying brain mechanisms remain unclear, we may hypothesize, in DM1 brains, a coexistence of two different mechanisms: one related to neurodevelopmental mechanisms, which might explain the positive correlations; the other one due to neurodegeneration, which might explain the negative correlations found between CTG triplet expansion size and cortical thickness. The present data are not exhaustive, and further studies are required to increase our comprehension of these possible concomitant phenomena.

Cortical thickness was also found being associated with various measures of SC in our DM1 patients. Positive correlations were indeed found between a correct identification of sadness and cortical thickness in the superior temporal gyrus bilaterally, in the right angular and precentral gyri, and in the left medial frontal gyrus. The medial frontal gyrus has been previously reported being involved in the integration of emotions into cognitive decisions and planning (42), whereas the angular gyrus is known to be involved in various cognitive functions through integration of information from different modalities. The angular gyrus is structurally connected to several brain areas including the frontal and parietal regions and the insular cortex. Against this background, we hypothesize that a reduction of cortical thickness in the angular gyrus might be responsible for its disconnection with a set of regions involved in emotion processing (43–50) and might therefore account for deficits in emotion recognition. Moreover, we found negative correlations between cortical thickness of the precuneus and the severity of abnormal behavior in social situations (51–53). This anatomical localization of abnormalities might account for difficulties in social judgment that we documented, for the first time, in our cohort of DM1 patients. Although the emotions are traditionally considered as modulated by the limbic system, there is evidence that the different aspects of emotions (i.e., recognition, attribution, monitoring) rely on the integrity of a widespread cortical network that includes the frontal areas [see Dixon et al. (54) for a review] and the cingulate cortex as part of the limbic system (55). In turn, these areas are known to interact with the premotor cortex and the limbic system [see (56)]. Additionally, the present study supports the idea of a diffuse involvement of these same brain areas in the attribution of emotions, as well as in more complex behaviors related to moral judgment. In this framework, the current work posits the recognition of “others' mental states” in DM1 patients as depending on complex brain networks.

However, in a previous study, Caso et al. (7) suggested that white matter changes in DM1 might be of neurodevelopmental origin, whereas gray matter changes are most likely due to neurodegeneration. Consistently with this view, we hypothesize here that both cortical thickness changes and their relationship with SC deficits might be due to neurodegeneration phenomena.

The main limitation of the present study is the relatively small sample size. Nevertheless, the current results are consistent with those obtained by other authors who investigated the ToM (57) and emotional recognition (19) in independent cohorts of DM1 patients. Further studies are, however, needed to confirm these findings and clarify whether different patterns of impairments may be associated to different clinical onsets of DM1.

In conclusion, although the small sample size and lacking of a wider knowledge on this topic make the present findings speculative, the present study shows the presence of diffuse SC deficits in DM1 patients. These deficits include also emotion processing and social judgment abilities. Moreover, the current study documents the association between emotional impairment and a pattern of brain damage involving the microstructural organization of many cortical regions in DM1 patients. These cortical abnormalities were correlated with the peripheral CTG triplet expansion size of our DM1 patients, thus reinforcing the idea that DM1 is a disease characterized by remarkable higher-level dysfunctions that may become clinically relevant over the clinical course (58). Nevertheless, there are many environmental factors that might contribute to the development of SC deficits in DM1. The cross-sectional design of the current study makes any causative inference between brain tissue abnormalities and symptoms observed in DM1 patients purely speculative. Further studies with a longitudinal design are needed to address these issues.

## Data Availability Statement

The datasets generated for this study are available on request to the corresponding author.

## Ethics Statement

The studies involving human participants were reviewed and approved by Local Ethical Committee of Santa Lucia Foundation, Rome Italy. The patients/participants provided their written informed consent to participate in this study.

## Author Contributions

LS conceived the idea of the study, coordinated the collection of cognitive and MRI data, supervised cognitive and MRI data analyses, and wrote the manuscript. GB run cognitive and MRI data analyses and wrote the manuscript. MBr conceived the idea of the study. GG implemented data analyses pipeline. CD and SB collected cognitive measures and MRI data. APet and GS patients' enrolment and supervised the draft. GS patients' enrolment and supervised the draft. APer patients' enrolment. GM and CC supervised the draft. MBo conceived the idea of the study, supervised the writing of the manuscript.

### Conflict of Interest

The authors declare that the research was conducted in the absence of any commercial or financial relationships that could be construed as a potential conflict of interest.
